# Mothers’ accounts of the impact on emotional wellbeing of organised peer support in pregnancy and early parenthood: a qualitative study

**DOI:** 10.1186/s12884-017-1220-0

**Published:** 2017-01-13

**Authors:** Jenny McLeish, Maggie Redshaw

**Affiliations:** Policy Research Unit in Maternal Health and Care, National Perinatal Epidemiology Unit, Nuffield, Department of Population Health, University of Oxford, Old Road Campus, Headington, Oxford, OX3 7LF UK

## Abstract

**Background:**

The transition to parenthood is a potentially vulnerable time for mothers’ mental health and approximately 9–21% of women experience depression and/or anxiety at this time. Many more experience sub-clinical symptoms of depression and anxiety, as well as stress, low self-esteem and a loss of confidence. Women’s emotional wellbeing is more at risk if they have little social support, a low income, are single parents or have a poor relationship with their partner. Peer support can comprise emotional, affirmational, informational and practical support; evidence of its impact on emotional wellbeing during pregnancy and afterwards is mixed.

**Methods:**

This was a descriptive qualitative study, informed by phenomenological social psychology, exploring women’s experiences of the impact of organised peer support on their emotional wellbeing during pregnancy and in early parenthood. Semi-structured qualitative interviews were undertaken with women who had received peer support provided by ten projects in different parts of England, including both projects offering ‘mental health’ peer support and others offering more broadly-based peer support. The majority of participants were disadvantaged Black and ethnic minority women, including recent migrants. Interviews were audio-recorded and transcripts were analysed using inductive thematic analysis.

**Results:**

47 mothers were interviewed. Two key themes emerged: (1) ‘mothers’ self-identified emotional needs’, containing the subthemes ‘emotional distress’, ‘stressful circumstances’, ‘lack of social support’, and ‘unwilling to be open with professionals’; and (2) ‘how peer support affects mothers’, containing the subthemes ‘social connection’, ‘being heard’, ‘building confidence’, ‘empowerment’, ‘feeling valued’, ‘reducing stress through practical support’ and ‘the significance of “mental health” peer experiences’. Women described how peer support contributed to reducing their low mood and anxiety by overcoming feelings of isolation, disempowerment and stress, and increasing feelings of self-esteem, self-efficacy and parenting competence.

**Conclusion:**

One-to-one peer support during pregnancy and after birth can have a number of interrelated positive impacts on the emotional wellbeing of mothers. Peer support is a promising and valued intervention, and may have particular salience for ethnic minority women, those who are recent migrants and women experiencing multiple disadvantages.

## Background

The perinatal period and transition to parenthood is a vulnerable time for mothers’ mental health. Approximately 9–13% of women experience depression at some time during pregnancy [1–3] and approximately 13–15% experience anxiety during pregnancy [4, 5]. Approximately 13–21% of women experience depression at some time in the year after birth [2, 6] and approximately 13% experience anxiety in the year after birth [4]. Women are more likely to experience antenatal and postnatal depression and anxiety if they are socially isolated and perceive themselves as having low social support, if they are single parents or have a poor relationship with their partner, if they have low self-esteem, if they are poor, or they are under 18 [7–10]. In addition to the impact of these mental health problems on the mother’s quality of life, there is evidence that the mother’s poor mental health both before and after birth can adversely affect her baby’s physical [11], psychological [12, 13], mental [14], emotional and behavioural [15] development, particularly in socio-economically disadvantaged families [16].

Because lack of social support is a significant risk factor for perinatal depression and anxiety [7–10], one intervention used to assist mothers with or at risk of perinatal mental health problems is peer support, described by Mead and MacNeil as being in general “defined by the fact that people who have like experiences can better relate and can consequently offer more authentic empathy and validation” [17]. Social support generally, and peer support specifically, are often described as comprising emotional, appraisal (affirmational), informational and sometimes instrumental (practical) support [18, 19]; and Leger and Letourneau argue that “peer support offers a fifth dimension of empathetic support” [20].

One peer support intervention for postnatal depression is to bring affected women together in support groups where they can feel ‘safe’ to talk about their feelings of distress, whereas outside the support group they may become isolated with their difficult emotions because of shame at having ‘failed’ at an idealised version of motherhood [21]; there is, however, no high quality evidence of lasting impact of peer support groups on symptoms of depression [22]. A second model of peer support for postnatal depression is telephone support from a briefly trained volunteer who has herself recovered from the condition, which has been reported as effective in preventing postnatal depression among women who are at high risk of developing it [23], and potentially in assisting recovery in women who have depression [24]. A third model is one-to-one visits from trained volunteers (who may or may not themselves have experience of mental health problems). The evidence of effectiveness is mixed. A small pilot randomised controlled trial found weekly peer support visits to be effective in reducing symptoms of postnatal depression as measured by the Edinburgh Postnatal Depression Scale [25], and a before and after study found volunteer visits to be associated with reduced anxiety and depression [26], but a cluster randomised study found that one-to-one volunteer visits did not prevent the onset of postnatal depression in women considered ‘at risk’ [27].

Many pregnant women and new mothers who do not have a diagnosed mental illness experience sub-threshold symptoms of depression and anxiety as they adapt to their maternal role [9], or stress, which can in itself adversely affect the developing baby [28]. It is also common for new mothers to experience low self-esteem and feelings of inadequacy when encountering discrepancy between their socially-conditioned expectations of motherhood, and its challenging reality [29, 30]. Less is known about the impact of receiving volunteer or peer support on these women’s emotional wellbeing more broadly, and the evidence is mixed. One randomised controlled trial showed that monthly postnatal visits from a minimally trained “community mother” could improve mothers’ self-esteem [31], while another randomised controlled trial found no impact on maternal mental health at one year [32]. There is as yet little qualitative research on women’s subjective experience of receiving perinatal volunteer or peer support outside the context of depression and in the antenatal as well as postnatal period. However, there is some evidence that regular visits from a trained volunteer doula (a woman who supports other women during pregnancy and birth) can help disadvantaged pregnant women and new mothers to feel less isolated, less unhappy, less afraid of birth and more confident [33, 34]. There is also some evidence that mothers who receive home visits from volunteers in postnatal programmes feel that having someone to talk to improves their emotional wellbeing [35–38], reduces stress [39], and makes them feel better about themselves and their parenting [40].

Aiming to fill this gap in the qualitative literature, this paper reports original qualitative research, carried out in England, that explores mainly disadvantaged and migrant women’s views about the impact of organised peer support on their emotional wellbeing during pregnancy and after birth, and their understanding of the mechanisms involved. It also considers whether these impacts and mechanisms, as identified by the mothers themselves, differ according to whether the mother and peer supporter have experience of a diagnosed mental health condition.

## Methods

### Study design

Because the purpose of this study was to explore mothers’ own perceptions and lived experiences, without generating or superimposing theory, an experiential qualitative descriptive design was chosen [41, 42], based on semi-structured interviews, and informed by the theoretical perspective of phenomenological social psychology [41]. This “low-inference” design [42] allows mothers’ voices to be heard while acknowledging the role of both participants’ understandings and the researchers’ interpretations in the production of knowledge [43].

### Participant recruitment

A researcher first contacted the co-ordinators of 10 peer support projects providing perinatal peer support in Bradford, Bristol, Burnley, Huddersfield, Halifax, Hull, London and rural North Yorkshire, to gain an understanding of the individual projects and to describe the research aims and process. Two projects specifically targeted ‘mental health’ and employed women with experience of perinatal mental illness to offer mothers counselling or more general peer support. Eight other projects were broadly-based, training unpaid volunteers, almost all of whom were mothers, to support a range of target groups: mothers living with HIV, mothers with very complex needs, young mothers, South Asian mothers, refugee and asylum seeker mothers, and mothers from a defined geographical area (but with a focus on disadvantaged women); the projects are shown in Table 1. Full details of the volunteer-based projects, some information about training and the key elements of the peer support provided have been reported elsewhere in an earlier paper where the objective was to describe the different models and key features of volunteer peer support currently in use [44]. These included active listening, providing information, signposting to local services and providing practical support.

**Table 1:** The peer support projects

	Primary target group	Location	Type of peer support	Period of support	Initial Training for volunteers
1	Women with very complex needs	London	Small team of volunteers	Pregnancy, at birth, to 12 weeks postnatal (longer if needed)	40 h
2	Women in local area	London	1:1 volunteer	Pregnancy to 3 months postnatal (longer if needed)	36 h
3	First time mothers in local area	London	1:1 volunteer	Pregnancy to 8 months postnatal	72 h
4	Women receiving maternity care from a specific hospital trust	London	1:1 volunteer	Pregnancy to 12 months postnatal	8 h
5	Refugees and asylum seekers	North of England	1:1, 2:1 volunteers	Pregnancy to 2 years postnatal	30 h
6	Young women and women experiencing difficult circumstances	North of England	1:1 volunteer, groups	Pregnancy to 2 years postnatal	30 h
7	Women from minority ethnic communities; young women	North of England	1:1 volunteer, groups	Pregnancy to 2 years postnatal	30 h
8	Women living with HIV	London	1:1 volunteer, group	Pregnancy and short period postnatal; 6–12 visits	36 h
9	Women with depression/ anxiety	West of England	1:1 paid peer supporter or volunteer, group	Pregnancy and postnatal. Group has 12 sessions; 1:1 for 6 weeks (more if needed)	In development
10	Women with depression/ anxiety	North of England	1:1 counselling from paid peer counsellors, drop in group	Pregnancy and postnatal, as required	Peer counsellors trained in counselling

The project co-ordinators described the research to supported mothers using the study information leaflet and either asked permission for the researcher to contact them, or arranged with those who wished to participate a time for interview. One mother decided not to participate when contacted by the researcher, citing logistical problems of caring for her baby.

### Data collection

The researcher met the women who agreed to participate, and obtained written informed consent before carrying out a face-to-face semi-structured interview based on the topic guide. Each interview explored the mother’s experiences of using the maternity services; how she heard about and decided to take up the peer support; the nature of the support and what she felt its impact had been; whether she felt there was any difference between receiving support from a volunteer and from a professional; how she felt about the ending of the support; and whether she would recommend any changes to the peer support. The duration of interviews varied (range 16–90 min, median 44 min); the shorter length of a few interviews was due to mothers needing to attend to their young children. Professional interpreting for participants whose first language was not English was offered, however, none took up the offer, although at the mother’s request one interview was informally interpreted by a peer supporter. All the interviews were audio-recorded and fully professionally transcribed.

### Data analysis

The mothers’ interviews were analysed using inductive thematic analysis [45]. Transcripts were first checked against the audio recording, and then read and reread, and codes were identified inductively and recorded using NVIVO software. Codes were refined, combined and disaggregated as data collection continued, and emergent themes identified; initial codes and emergent themes were reconsidered in the light of subsequent interviews using constant comparison [46]. To ensure the validity of the analysis, one researcher (JM) undertook thematic analysis of all the transcripts and the other (MR) analysed a subset. Codes and emerging themes were discussed and agreed. Both researchers were aware of the need to approach the analysis reflexively, putting aside their existing knowledge of the topic so that the analysis remained close to participants’ accounts, and acknowledging the potential impact of their own perspectives as White, UK-born women with children.

## Results

A total of 47 mothers who had received peer support during pregnancy and after birth took part in semi-structured qualitative interviews between July 2013 and September 2014. 46 interviews were carried out face-to-face and one interview was carried out by telephone at the mother’s request (oral informed consent was given and recorded in writing).

**Fig. 1:**
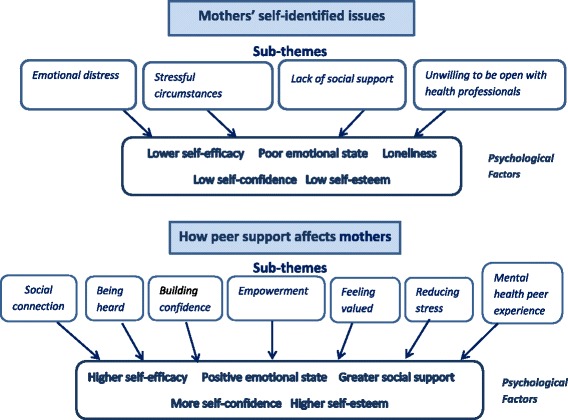
Two key themes and associated subthemes in women’s identification of their emotional needs, and the psychological factors involved in the peer support process that impacted on their wellbeing

This section describes firstly, the participants’ characteristics, and secondly, the results of the thematic analysis. There were two key themes: (1) ‘mothers’ self-identified emotional needs’ and (2) ‘how peer support affects mothers’. These key themes and their associated subthemes are shown in Fig. 1.

### The participants

The 47 mothers ranged in age from 19 to over 40. Seven were supported by the two ‘mental health’ projects and 40 were supported by the eight ‘broadly-based’ projects. Twenty seven were first time mothers (including one currently pregnant with her first child), ten had two children, six had three children, two had four children and two had five children. One was a grandmother with legal care of her grandchild. Twenty six were single parents (without a partner) but only one had actively chosen single parenthood. One mother was living apart from all five of her children and a further five had left older children behind in their home country. Thirty one were born overseas, coming from Africa, Eastern Europe, South America, South Asia, East Asia, the Caribbean and the Middle East; the most common ethnicity for these migrants was Black African (17 mothers). Thirty did not speak English as their first language. Of the 16 mothers born in the UK, one was Asian, two were Black and one was White. All of the mothers supported by the ‘mental health’ projects were White British.

The mothers interviewed had experienced a range of traumatic experiences before pregnancy, including forced migration, having children taken into care, and the death of a child or partner. Ongoing stressful experiences during pregnancy and afterwards included unemployment, poverty, homelessness, chronic ill health, domestic abuse, children with health or behavioural problems, and insecure immigration status.

### Mothers’ self-identified emotional needs

This key theme reflects mothers’ descriptions of their needs and the factors which they saw as affecting those needs. Four subthemes emerged: ‘emotional distress’, ‘stressful circumstances’, ‘lack of social support’, and ‘unwilling to be open with professionals’.

#### Emotional distress

This subtheme relates to mothers’ own descriptions of their distress. Three quarters of the mothers identified themselves as suffering from depression, anxiety or panic attacks during pregnancy or after birth (including all the mothers supported by the ‘mental health’ projects and two thirds of the mothers supported by the ‘broadly-based’ projects), although only half of these said they had received a formal diagnosis. Five mothers described having suicidal thoughts during pregnancy or afterwards (one in a ‘mental health’ project and four in ‘broadly-based’ projects): ‘*I crashed really badly. To the point where I was feeling suicidal*’ (M042). Some described specific anxieties focused on miscarriage, birth or the baby’s health: ‘*I felt quite anxious about the birth and being out of control… In my mind was all about, “It can only go wrong*”’ (M015); ‘*I was constantly thinking, “Is she still breathing? Is she still breathing? Hasn’t she forgot to breathe?*”’ (M008).

Many mothers described a collapse in their self-confidence when they compared themselves unfavourably to idealised images of ‘perfect’ motherhood: “*I completely lost my confidence that time…Always there is a feeling in the back of my head that “Am I doing it right? Am I a good mother?”*’ (M002), and for some this included losing social confidence because of fears of being judged by other mothers: ‘*I became a little bit withdrawn… I didn’t want to go into details and open up to complete strangers, ‘cause I didn’t want them to judge m*e’ (M019). For some mothers experiencing mental illness, feelings of inadequacy had precipitated a loss of self-esteem and the development of a negative maternal identity: *‘I felt like I’d lost my identity… I was just convinced that I was the world’s crappest mum’* (M038); “*You feel like you’re not doing a good enough job as a parent. And I used to feel like I was failing*” (M029).

#### Stressful circumstances

This subtheme reflects mothers’ accounts of the emotional effects of their difficult life experiences. Almost all the mothers referred to ways in which current difficult circumstances had affected their emotional wellbeing during pregnancy or afterwards, saying that their situation made them feel ‘*stressed*’, ‘*sad*’, ‘*scared*’, ‘*afraid*’, ‘*frightened*’, ‘*desperate*’, or ‘*cursed*’. Some also said explicitly that past traumas continued to weigh on their minds: ‘*Every time you’re asleep you dream about [the gang rape], the flashback, it’s killing. I don’t pray for anybody to be in that situation when they are pregnant*’ (M033). Several mothers described how they had simply felt overwhelmed by their situation and lacked the resources to cope, feelings consistent with low self-efficacy: ‘*And it was the whole world is upon me… How am I going to cope?’* (M028); ‘*I am struggling to survive, I am struggling to look after myself and my kids…sometimes I feel old and beyond my ability’* (M036). One mother had fantasised about going to sleep until her postnatal depression had passed: “*I just didn’t want to be there anymore… there was times when I’d look at a bus and think, ‘If I could just get run over and go in hospital, even for a few months, I’ll wake up and then I’ll be okay”*’ (M038).

#### Lack of social support

This subtheme considers how all the mothers described their lack of meaningful social connection. Most of the mothers were in situations where they had considerable unmet needs for social support, as defined by Brown (cited by Oakley): “information, nurturance, empathy, encouragement, validating behaviour, constructive genuineness, sharedness and reciprocity, instrumental help, or recognition of competence.” [18] Their sense of social isolation had a number of components, explored below.

Many mothers felt physically alone. Thirty one were migrants to the UK, including nine who had arrived seeking asylum or were victims of people trafficking. They commonly had limited social connections in the UK and some had no connections at all: ‘*I don’t even know anybody here, I don’t know where to start*’ (M021). Some mothers had not had time to build a local social network because they had been dispersed under the asylum support system to unfamiliar places or had experienced homelessness and the frequent moves associated with living in temporary accommodation: *‘We lived in [one area], and then I had to move to [another area], and then Council again moved me here… We don’t have any friends here. Only by ourselves’* (M001). Some found it hard to make friends in the transient community on an Army base: ‘*Everybody seems to be, keep themselves to themselves… I’ve been housebound basically. Just don’t know anyone’* (M023), despite their efforts to join local groups: *“I’ve walked into the room and everyone’s already got their friend groups and they don’t want to talk to you”* (M027). Many mothers spoke about how this physical isolation engendered loneliness: ‘*Lonely…sometimes [my husband] would be the only person I’d see for weeks, that I’d actually talk to*’ (M026).

Some mothers might have appeared to have social support around them, but said they felt unable to share difficult thoughts and feelings with family and friends, for a range of reasons. Some mothers had a partner who was unsupportive or abusive: ‘*He’s very focused on himself…it wasn’t good for my self-esteem at all ‘cause he really was cruel to me*’ (M039). Some said it was because family and friends would respond with criticism or inappropriate advice, or because it would feel like an admission of failure: ‘*Your friends, your family are telling you, “You must do this, not do that”* (M043); ‘*I can’t tell people I can’t cope. In Africa they would say “Then why did you get pregnant?”* (M006). For others it was because their family were too emotionally invested and would deny the validity of the mother’s concerns or would be upset by her feelings: ‘*Sometimes [my husband] says, “No, it’s in your mind”. But it’s not always in my mind…I am always worried. I didn’t tell this to my husband or my mum, I thought maybe they would feel more worried’* (M002). Some mothers with diagnosed mental health issues concealed their feelings because they found friends and family judgemental about mental illness, or felt ashamed and guilty that they were not enjoying motherhood: *‘I pretend to be happy, I do that with my family as well ‘cause I haven’t told them [about my depression]…They don’t react nicely’* (M040); *‘[People say,] “I don’t understand how she can be depressed when she’s just had a baby, one of the most beautifullest things in the world” …That makes you go even more into your shell and feel more embarrassed and distraught…so ashamed’* (M038).

Some mothers were estranged from family, friends or community, because they had transgressed cultural norms: *‘We are Muslim and my mum she’s like strict, and [for] the woman to be pregnant, she have to be married first…when I was pregnant three months she told me I have to go away’* (M010). Some young mothers had found their friends had lost interest in them: ‘*I lost most of my friends really when I had a baby…People who are my age without kids, they just want to go out all the time drinking and stuff whereas I’m not like that obviously’* (M027). Other mothers had deliberately isolated themselves because they felt vulnerable to gossip and criticism: *‘We had very dense community and everybody know each other and they talk…and they put their nose in every issues…they make gossip.. And then day by day I stopped with my friends’* (M036).

#### Unwilling to be open with professionals

This subtheme describes how most mothers did not feel able to make use of professional help for their feelings of emotional distress. Many extended the self-censorship which they practised with family and friends, to their interactions with health professionals. For example, midwives were consistently said to be too busy to listen to women’s concerns: *‘If you are at the hospital, midwife [is] limited. I can’t really ease my worry [to] those people’* (M046); and to have a professional agenda that did not include meaningfully addressing emotional needs:

*‘[The midwives] were all really nice… but I feel they actually had their own agenda…The checklist – “Blood pressure, is it fine? Are we having the urine test? And let’s feel the baby.” So they do ask, “Oh how are you feeling?” But that’s very much at the bottom of the priorities… they don’t have the knowledge to actually deal with it’.* (M015)

Several mothers with diagnosed mental health problems distrusted mental health and social care professionals, who they felt lacked empathy or genuine interest: ‘*It’s just their job. So just they’re not really interested in what I’ve got to say or they’re not really bothered’* (M041). They were guarded in their interactions with these professionals who had, they believed, stereotyped responses to people with mental health problems: ‘*If you ring social services …well I don’t dare ask for any help, [their only response is] child protection, child protection, child protection’* (M040); and were primarily watching them for signs of failure: ‘*[The perinatal psychiatric team] were there just to observe me…I always felt like they are picking up on what I am not doing right’* (M006).

### How peer support affects mothers

This key theme reflects women’s accounts of the impact of receiving peer support during pregnancy and after birth on their emotional wellbeing. Seven subthemes were identified: ‘social connection’, ‘being heard’, ‘building confidence’, ‘empowerment’, ‘feeling valued’, ‘reducing stress through practical support’ and ‘the significance of ‘mental health’ peer experiences’. Figure 1 illustrates how they relate to the mothers’ self-identified needs. There were no identifiable differences related to mothers’ socio-demographic characteristics in the way that they described these impacts.

#### Social connection

This subtheme examines how, for the mothers without accessible social networks, visits and calls from their peer supporter were in themselves an important source of morale-boosting social contact: *‘If [the peer supporter] wasn’t there I would feel like alone, crying every day’* (M010). Many of the peer supporters also accompanied mothers to local parent groups where they could meet others, and which they had lacked the confidence to attend alone. In four projects the peer supporters ran their own discussion or activity groups to bring mothers together for mutual support, increasing women’s sense of social connection and confidence: *“It’s made me a lot more confident because I’m getting out more and I’m seeing people more”* (M025)*; “A lot of mothers with depression just feel like they’re alone, and when I got [to the group] I didn’t feel alone anymore”* (M029).

A few women described how accepting the peer support felt like taking a risk because it might impose an additional stressful social obligation, but in all cases they were quickly won over by positive experiences of the peer support relationship: *‘I just thought, “It’s going to be somebody that’s going to come round every other day and do my head in … [But] I stuck with it and… I’m glad I actually got the support because they are actually like really, really friendly’* (M020).

There were some examples where a peer support project offered a fairly structured programme of (non-emotional) mentoring, but where the supported women nonetheless described the support primarily in terms of the emotional connection they derived from it: ‘*I found so lovely and nice…[the peer supporter] come here and we talk together. A bit, I feel I have someone to talk to’* (M036). However, one mother expressed the view that having monthly ‘mentoring’ visits was insufficient for a strong relationship to develop: ‘*I would like to have seen [the peer supporter] a bit more often… you don’t really develop a huge relationship’* (M003).

#### Being heard

Across all of the projects, peer supporters had been trained in non-directive listening and this was the aspect of their support that was most frequently mentioned by the mothers, who described the emotional release of being able to talk openly, particularly about their feelings of emotional distress: ‘*When the problem is really, really much I feel depressed, I just call her and she listens to me. I just smash everything on her and she listens to me’* (M028). By contrast with mothers’ experiences with family and friends, the peer supporter listened without passing judgement or giving advice, and accepted the mother’s feelings: *‘You can be open and you can be yourself, and if you have got something on your mind you know you can say it without being judged’* (M019). Mothers who felt stigmatised by their situations found it a relief to be able to confide in volunteers who were outside their normal social circle, and were bound by a duty of confidentiality:

*‘The circle of people that you are around, some things you can’t share with them because they will go and tell other people…so I’ve been keeping things inside me for a very long time. So when I get to meet [a peer supporter] where I can share things with, it feels like there’s a burden lift off my shoulder.’* (M012)

Many mothers juxtaposed what they saw as midwives’ superficial invitation to disclose feelings with the real emotional support from their peer supporter, who had built up a relationship of trust with them. Some said that because of this relationship, the peer supporter was the first person with whom they had been able to be honest about how they felt: ‘[*The peer supporter] was someone who talked to me all the time, kept in touch with me all the time, so if someone is talking to you, is building that kind of relationship, you kind of feel confident to share with them anything’* (M045). One mother contrasted her disappointment at confiding in a midwife who did not continue to provide antenatal care, with her ongoing relationship with a peer supporter: ‘*My midwife know about my situation… [Then] I couldn’t see her anymore.… I was trusting her, I tell her all my life, and then later she showed me her back…[The peer supporter] was good…just like a sister’* (M014).

Many of the mothers who had a diagnosed mental health condition described their relief at being able to talk openly about their mental health to a peer supporter, compared with unsupportive family or friends: ‘*[Your family] might think you’re perfectly fine… but you feel like you’re going absolutely to bits … Having [a peer supporter] to talk about your issues with every week is a big release’* (M039). They also contrasted what they found to be the off-putting attitudes of health and social care professionals with the positive attitude of their peer supporters, both those who had their own experiences of mental health problems and those who did not:

‘*[The peer supporters] really supported me wholesomely to make sure that I went through the whole process okay… whereas in the hospitals or the midwives I always knew whatever I say to them will be on my notes’*(M006, broadly-based project)

*‘I thought if I said something I would get Social Services back and they’d come and take [my baby]…You can talk to [the peer supporters] about anything and they’ll try and help if they can…they’re more supportive, down-to-earth, caring.’* (M040, mental health project)

#### Building confidence

This subtheme considers the ways in which peer supporters helped mothers to rebuild their self-confidence. Peer supporters in the ‘broadly-based’ projects consistently gave the mothers positive feedback and focused on their strengths: ‘*[The peer supporter] made me feel better because [she was] speaking always good things [about] me’* (M013). They also challenged mothers’ self-perception that they were abnormal or inadequate, by normalising their parenting concerns: ‘*[The peer supporter] gave me the confidence…the first thing she said to me was, “You’re doing OK and this is normal”’* (M003). In some cases peer supporters achieved this by drawing on their own lives as mothers to demonstrate that becoming a parent is a learning experience for everyone: ‘*[The peer supporter] talk about…her personal experience. Or how she look after her kids, and that’s made me a bit calm and I say, “Oh my gosh, just not me I have this difficulty. People had before”’* (M036). Where the peer supporters led groups for the mothers, members of the group could provide this validation of feelings and experiences: *“It’s nice to know that other people have felt the same…you feel like it’s normal to have those feelings rather than just be sat at home thinking you’re the only one feeling like that”* (M027). Where the group was skilfully led and structured inclusively to avoid the social cliques that mothers had experienced in drop-in groups, this also enabled mothers to succeed at social interactions and increased their social confidence: “*When you see that they are actually interested in what you’re saying… that’s made me a bit more confident that I should just be a bit more positive about speaking to people and not just think they don’t want to talk to me*” (M027).

Mothers at the ‘mental health’ projects described the beneficial dynamic when their peer supporters talked about some of their own experiences of mental illness and offered non-judgemental acceptance of women’s difficult feelings: ‘*You’ve got the acceptance here and it kind of gives you a bit of acceptance of yourself…It’s like unconditional love really’* (M039). Peer supporters also role-modelled the pathway to recovery, which inspired mothers with confidence about their future: ‘*Everything that [the peer supporters] used to say, I felt like I could trust in that, because I could see that they were well. [They’re] living proof’* (M038). This normalisation of their mood and experience was particularly important for the self-confidence of those depressed or anxious mothers who struggled with a sense of profound failure when they compared themselves with other women who appeared to be succeeding effortlessly at motherhood:

*‘I used to feel like everyone was watching me … like they’d all be judging me…[Other women] made it look so easy, and their babies were just so well behaved and they all looked so perfect…and I was just struggling just to get out the house in the morning…I always looked a mess and I just used to feel really sweaty and minging all the time… I knew that [the peer supporter]’d understand 'cause she’d been through it.’* (M038)

As for mothers in the ‘broadly-based’ projects, the groups run by peer supporters for mothers with mental health problems could also provide a powerful experience of normalisation: “*To be able to come and not feel different…. This was like my safe haven for two hours a week”* (M038); they were also places where women could be honest about their feelings: ‘*Everybody else is in the same boat so you can talk to them about [depression] and they don’t criticise you like other mums do. Made me feel more confident… I can succeed’* (M040). For one mother with anxiety, however, the group environment was more than she could cope with: *‘It was just awful… it was too overwhelming. I felt too many strong overpowering feelings’* (M039).

#### Empowerment

This subtheme looks at how mothers felt empowered by the informational, motivational and moral support aspects of peer support, and how this boosted their self-efficacy in the face of serious challenges. In most projects, an important part of the peer supporters’ role was enabling women to find solutions to their problems and to make informed decisions about their maternity options and other issues. They did this by offering evidence-based information or signposting to reliable sources of information, and then helping the mother to reflect on the different options and come to her own decision. The peer supporter training in most projects strongly emphasised the importance of giving non-directive information rather than ‘advice’ (even if the mother asked for advice), so the mothers remained in control of their decisions: ‘*[The peer supporter]’ll never give you the answers, she’d just suggest stuff… she’ll say, “Have you tried this, have you tried that?”’* (M003). Another aspect of empowerment was orientating women around their communities so that they understood the services available to them locally, and were thus able to resolve some of the stressful practical issues they faced: ‘*Lots of people [are] there for your help but if you don’t know, you can’t get any help…When you have [a peer supporter] they have contact with everywhere’* (M011).

The mothers described the impact of this empowering approach as reducing their anxiety and making them feel more in control:

*‘The first time I met my volunteer she asked me what’s my biggest concern. At that time my biggest concern is, I will give birth on the way [to hospital]…The volunteer did not laugh [at] me, she just helped me to find some information from books, from internet, that discuss whole three stage of labour. Then I feel ready to understand what will happen.’* (M043)

For some mothers with low self-efficacy, the peer supporters’ unconditional support and affirmation gave women a renewed sense of agency over their lives:

*‘[The peer supporter] used to ask me, “What do you want to do? You can do it. I know you can do it.” I have always been living toward the things we wrote down…I’m more in control of my life, I know what I want and I’m going for it.’* (M006, broadly-based project)

*‘[With] a health professional…you’re not in control, you have to come to them for what’s wrong with you. When you come here… [the peer supporter] hasn’t got all these ideas about how I am and who I am and what I can do and what I can’t do….she doesn’t see any limits on what I can do.’* (M039, mental health project)

Even where they were unable to help with solving intractable social or legal problems which made some mothers feel trapped and powerless, some peer supporters offered mothers relief by being a supportive presence with solidarity and hopeful words, and where there was a shared religious faith, sometimes by praying together: ‘*You are able to pour all your stress out by talking to someone, even if they don’t have to tell you what to do…[The peer supporter] was really loving and she also helped me spiritually…she would pray with me’* (M045). Several of the Black African mothers described this moral and spiritual support as “encouragement” and said that it made them feel stronger and better able to cope with their problems: ‘*[The peer supporter] gave me courage… Sometimes when you feel yourself lonely and you are down, [if] you have somebody [to] encourage you, so that is the difference, it’s not like you are alone’* (M014).

#### Feeling valued

A consistent subtheme across all projects was how mothers experienced the peer supporters’ relationship with them as strongly contributing to increased feelings of self-esteem. The mothers felt that the peer supporters cared about them as individuals, and were unconditionally woman-focused rather than primarily interested (like statutory services) in their children:

*‘[The peer supporters] were about me…whereas everybody else was about [my baby] first and, well [I] don’t really matter… It was always about me and how I felt and what I needed to make me be able to be a better mum … And something that was nice, “Do you know what? You’re doing really well.”’* (M007)

For many of the mothers in the ‘broadly- based’ projects, the fact that their peer supporter was a volunteer was an important component in this feeling of being valued, because they perceived her as caring enough to give them her own time:

*‘The lonely life one lives in this country, I felt it was really heart-warming for someone to come in and see me and talk to me and find out how things are going…I come from Africa, so I know when someone comes to visit you, they’ve taken their time and they are just thinking about you.’* (M045)

In some cases, this consistent, long term peer support relationship had a transformative effect on the mother’s feelings about herself:

*‘Before I’d be like, “Oh, I don’t want to even get dressed.” … [The peer supporter]’s kind of boosted my confidence and self-esteem. Like now I’ll actually take time and … do my hair and do a bit of make-up and go out and look nice, and it’s like before I really couldn’t be bothered because I were constantly feeling low about myself.’* (M020)

Several of the mothers described how having a peer supporter alongside them was an emotional lifeline when they felt completely alone in their difficult situation and were having suicidal thoughts - the consistent, caring contact broke through their sense of isolation and despair:

*‘The whole stress was just too much, everything was heavy on me… During that time I was thinking, “It’s the end”…[The peer supporters] didn’t allow me to think I don’t have anyone, nobody to look after me. I can see a brighter future now.’* (M004)

However, for one vulnerable mother contact with a peer supporter had left her feeling undervalued because she felt that her concerns were not taken sufficiently seriously (the project then allocated her a different peer supporter):

*‘I felt [the] volunteer was trying to put me down, like when she made some calls to some charity for help and I did a follow-up call to say, “So what happened?”, she was like, “Oh it was just the other day, you know, I’ve got other people [to support].”’* (M034)

Although most of the projects offered support for a defined period (ending at between 6 weeks and two years after birth), in many cases the supporters remained informally in contact with the women they supported after the end of the ‘official’ support. Where this occurred it reinforced the mothers’ belief that the peer supporters had a valued and real relationship with them and were not just offering a ‘service’: ‘*We staying friend, even yesterday I was spoken to them, so we still alright…It was, “How are you? How are things going? When I’m going to see you now?” Like friends’* (M014).

#### Reducing stress

Many of the projects offered practical support such as second hand baby clothes and equipment, or help with fares to hospital; and some individual volunteers spontaneously gave mothers help with shopping and cooking after the baby was born, transport, interpreting, or looked after the newborn baby for a short time so that the mother could rest. Mothers who had received practical support usually said that this was as important to them as emotional support, and some said it carried an emotional meaning: ‘*[The peer supporter] was encouraging. Not only with words…When I am stressed, the way she would make food for me, it has given me encouragement’* (M028). Some women explicitly described how the support affected their emotional wellbeing by reducing anxiety about practical problems:

*‘Everything was just sort of either black or white, and there were no grey areas. So if you don’t have a house you have nowhere to sleep, if you don’t have clothes the child won’t have anything to dress in. And that would give me a lot of stress. [The peer supporters] assured me they are going to provide me with the first clothes she would wear, so that really settled me up, so I’m thinking, “Really I don’t need to worry.”’* (M006)

#### The significance of ‘mental health’ peer experience

Most of the mothers supported by the ‘mental health’ projects believed that the shared experience of mental illness underpinned the effectiveness of peer support by creating a safe space for self-disclosure and inspiring hope for recovery: ‘*Talking to someone who’d gone through [postnatal depression] made me feel okay about divulging some of the things that I was thinking and feeling’* (M038). However, one mother took the view that shared experience was helpful but not essential, and that the most important quality of a supporter was the right attitude: ‘*As long as you’ve got that personality behind you, like to sympathise with the person and you want to help, then I think there is people [without depression] that can help others with depression*’ (M029).

This view was implicitly supported by the two thirds of the mothers in the ‘broadly-based’ projects who identified themselves as suffering from depression, anxiety or panic attacks, but whose peer supporter had not (necessarily) herself experienced mental health issues during pregnancy or afterwards. For almost all of these mothers, poor mental health was just one of the difficult issues in their lives. In some projects, the ‘peer’ element was conceptualised as based on a different issue (for example, shared experience of living with HIV, seeking asylum, being a young parent or having a shared cultural background.) In other projects there was no requirement of having a shared ‘issue’, and the ‘peer’ element came almost entirely from a shared experience of motherhood. All of these mothers nonetheless felt able to talk openly about their mental and emotional health to their supporter without fear of stigmatisation and said they felt better for having done so:

*‘When I got pregnant I got depression … start to cry all the time…. I was thinking, “How am I going to tell to the person which I never met what happened to me?” …[The peer supporter] said, “Don’t worry or anything, everything will be fine.” And she is saying that way that you believe her*.’ (M035, broadly-based project)

## Discussion

The impact of peer support was studied at a critical time in the lives of women who were commonly experiencing multiple disadvantages. Robust quantitative studies of one-to-one peer support are difficult to carry out, given the heterogeneity, relatively small scale and largely short-term funding of projects involved, and the difficulties inherent in trying to standardise the encounters between individual peer supporters and those they support. [47–49] This qualitative study adds to the literature by showing how mainly disadvantaged mothers experience and describe one-to-one peer support from a trained supporter, during pregnancy and after birth, as having a number of substantial and interlocking positive impacts on their emotional wellbeing.

Research on social support has consistently found that “a person’s perception of the availability of others as a resource contributes significantly to the individual’s self-regulation of distress” [50], and the absence of social support is a significant risk factor for antenatal and postnatal depression and anxiety [7–10]. It was notable that all of the mothers in this study were either actually highly socially isolated (the structural dimension of social support), or did not perceive themselves as having sufficient functional social support because they felt unable to confide in their partner, family or friends.

Beyond the basic human need for social connection, the most prominent theme in these interviews, was ‘being heard’ – mothers’ relief at having someone non-judgemental to talk with honestly about their problems, fears, concerns and other feelings. This emphasis on skilled listening is reflected in other studies that report mothers’ perceptions of the impact and benefits of organised peer or volunteer support. [26, 33, 35–37, 39, 40, 51, 52] ‘Being heard’ by a peer support volunteer or peer counsellor appears to have a comparable function to the ‘safe arena’ for connecting with others, sharing experiences, and ‘unsilencing voices’, identified by Jones et al. [21] as key to alleviating the burden of distress for mothers with perinatal mental health issues. It was striking that for some women who had experienced considerable adversity and were living in very difficult circumstances, it was a source of consolation and encouragement simply to have a peer supporter expressing moral support and solidarity, even if there was little she could do to improve the situation.

The mothers described a range of specific reasons why they concealed their thoughts and feelings in social situations and from health professionals, but not from peer supporters. Like the mothers interviewed by Tammentie et al. [53], they were unwilling to speak honestly about their feelings because they felt family and friends would not understand, would be upset and deny the validity of the woman’s feelings, or were likely to respond with inappropriate advice, criticism or gossip. Although health professionals are expected to ask women about their mental health history and their current emotional wellbeing at structured points during pregnancy and the postnatal period [22], many of the mothers had not felt able to discuss their feelings honestly in response to these questions. They characterised health professionals as too time-pressed to genuinely listen, focused on parenting deficits and safeguarding risks, likely to make assumptions, and interested in the baby but not the mother; the absence of continuity of care was also an obstacle. This finds strong echoes in Raymond’s study in the context of antenatal depression [54], where women identified barriers to disclosing depressive feelings to a midwife as having multiple caregivers during pregnancy, not being taken seriously, being rushed, and not being encouraged to talk. These potential missed opportunities for diagnosis are also reflected in midwives’ concerns about asking pregnant women about their mental health [55].

Meetings with the peer supporters over time changed mothers’ feelings about themselves. Consistent positive feedback (appraisal) and the peer supporters’ sharing of their own parenting experiences helped to normalise their concerns and build their self-esteem and self-confidence in their parenting role; while the peer supporters’ support for informed decision making increased feelings of self-efficacy and empowerment, reflecting the impact of volunteers found by Granville and Sugarman [34] and Spiby et al. [33] Like the mothers interviewed by Mauthner [56] and Letourneau et al. [57], the mothers in the ‘mental health’ projects described how comparing themselves to non-depressed mothers made them feel alienated and abnormal, and they had struggled with feelings of shame and motherhood failure. By contrast, receiving counselling or social support from workers who had experienced and recovered from perinatal mental illness, made them feel understood and accepted; they felt safe disclosing difficult thoughts and feelings and felt more optimistic about recovering when they met other women who had recovered (as reported by Mongomery et al. [58]), a process described by Jones et al. as “an essential aspect of the transition into maternal self-efficacy” [21].

The self-esteem of mothers in all the projects was enhanced by believing that their peer supporters genuinely cared about them, and that they had a real relationship (the nature of these relationships between women and their peer supporters is explored more fully in an earlier paper describing the different models of peer support used in the different projects [44].) Where the peer supporter was a volunteer, the support carried an additional emotional meaning: the mother’s worth was affirmed by the volunteer choosing to invest significant amounts of her own time in the relationship.

It is an inherent challenge in peer support to manage the ending of the relationship appropriately [32] [32] [32] and this may be particularly important when the peer support is offered during an emotionally intense life transition such as having a baby and withdrawn during the weeks after birth. In a study of support from volunteer doulas during pregnancy and birth and for 6–12 weeks after birth (with no further contact permitted), Spiby et al. found that a third of the women felt the support had ended too soon and “the feelings of loss associated with endings were identified as itself constituting an impact for women.” [33] By contrast, endings were handled less abruptly in the projects in this study, and some mothers described how their peer support had evolved into an enduring friendship, affirming the emotional validity of the original relationship.

It has been recognised that peer volunteers can potentially have negative impacts on those they support, for example, Dennis [51] reported that 10% of mothers receiving telephone peer support said that their volunteer had minimised their problems, possibly (the author suggests) in an effort to normalise their situations. There was only one report of a negative impact on emotional wellbeing in this study, with a mother who felt that her peer supporter had belittled her concerns.

When the accounts of the seven mothers who received ‘mental health’ support were set alongside the accounts of the forty mothers who received more broadly based support, clear parallels emerged. Under the subthemes of ‘being heard’ and ‘building confidence’, the experiences of mothers receiving support from ‘mental health’ projects were effectively amplified versions of the experiences of mothers supported by the other projects. Whether or not the mother had a mental health problem, the core issue was women feeling alone with their problems, emotionally and physically isolated, and not like other mothers. The core contribution of peer support to emotional wellbeing was to enable them to confide honestly in someone who listened unconditionally and affirmed their competence and value, either directly or by involving them in a group that could function constructively as an appropriate reference group for normalisation.

The seven women receiving ‘mental health’ peer support all described the specific and profound benefits of this support, consistent with previous research [21], but one challenged the notion that only people with experience of mental health problems could give effective support. This reflects the accounts of the other women who received support from projects without a specific mental health focus, many of whom described themselves as suffering from depression, anxiety or panic attacks. In spite of the lack of personal ‘mental health’ peer experience, these mothers all said they were able to talk openly to their peer supporter about their depression and anxiety, reporting an increased sense of emotional wellbeing as the consequence of the contact.

Leger and Letourneau [20] argue that an essential element of being a peer supporter is the shared experience and knowing what it is like to cope with and recover from postpartum depression. This is the definition of peer support as commonly used in the field of mental health [17]. However, for many of the mothers in this study, the ‘peer’ aspect of the support came simply from a shared experience of motherhood. For some, the ‘peer’ aspect was more specific, but did not arise in relation to mental health. Irrespective of the ‘issue’ by which the different projects chose to identify the recipients of peer support, the most important thing for mothers was that the peer supporter listened to the mother in the context of a warm, unconditional, non-judgemental relationship [59]. This suggests that having specific ‘peer’ experiences was an important mechanism for building trust for some vulnerable women, but that overall, attitudes were more important than circumstances in enabling mothers to speak honestly to the peer supporter about their feelings and to derive emotional support from the encounter. This is consistent with the findings of Letourneau and Secco [24], that many women with postnatal depression preferred one to one support from a professional or peer who had an understanding of symptoms and treatments, was non-judgmental, and ideally *but not necessarily* had experienced and recovered from the illness. It may thus be the case that, where pregnant women and new mothers are experiencing multiple disadvantages besides their mental health issues, careful selection and training of peer supporters is the key to providing effective emotional support, rather than necessarily the matching of women to supporters with specific mental health experiences of their own. Maternity care professionals should be sensitive to the possibility that a pregnant woman or new mother who may appear to be supported by a partner and local social network, may in reality be profoundly lacking in meaningful social support, and may therefore benefit from peer support.

This paper contributes to the literature through its dual focus on mothers with and without diagnosed mental health problems, and the participant groups included in this peer support study. A strength of the research was the use of in-depth qualitative interviews to explore the peer support experiences of 47 mainly disadvantaged mothers of very diverse multicultural backgrounds and with a range of challenging life experiences, whose voices are often not heard in research. Another strength was that the mothers were drawn from 10 peer support projects around England, enabling the experiences of mothers who received support from projects with and without a ‘mental health’ focus to be presented together. One limitation was that participants were contacted through the project co-ordinators - this was essential to gain the trust of some very vulnerable women, but meant that the researchers were not aware of how many declined to participate at that stage. One mother’s interview was informally interpreted by her peer supporter at her request, so her comments about the impact of the support she had received had to be considered in this context (she is not quoted in this paper). A further limitation was that, because of the possibility that women receiving some types of state support could be moved to another part of the country at any time, some mothers were interviewed sooner than was originally planned and had not yet experienced the ending of their peer support.

## Conclusion

Qualitative evidence from the study suggests that peer support can contribute to reducing low mood and anxiety by overcoming feelings of isolation, disempowerment and stress, supporting improvements in mothers’ feelings of self-esteem, self-efficacy and parenting competence. Identified benefits for maternal mental health and wellbeing indicate that peer support is a promising and valued intervention at a critical time in the transition to parenthood and may be particularly valuable for migrant women and women experiencing multiple disadvantages.

Care provision and funding for pregnancy and postnatal peer support projects should recognise the positive impact of receiving face to face, organised support from trained supporters. Further research could explore, both qualitatively and quantitatively, the extent and ways in which perceptions of peer support and its impact on emotional wellbeing differ for mothers from a range of different cultural and socio-economic backgrounds, with diverse and varying challenges in their lives, and with varying degrees of severity of mental illness.
